# Electroacupuncture at Zusanli Alleviates Sepsis by Regulating the TLR4-MyD88-NF-Kappa B Pathway and Diversity of Intestinal Flora

**DOI:** 10.1155/2022/6706622

**Published:** 2022-06-10

**Authors:** Lianghui Zhan, Hao Liu, Jingru Zheng, Jianbiao Meng, Danting Fu, Lisha Pang, Chunlian Ji

**Affiliations:** ^1^Tongde Hospital of Zhejiang Province, Hangzhou, Zhejiang 310014, China; ^2^Zhejiang Academy of Traditional Chinese Medicine, Hangzhou, Zhejiang 310014, China; ^3^Zhejiang Chinese Medical University, Hangzhou, Zhejiang 310014, China

## Abstract

**Background:**

Electroacupuncture (EA) at the Zusanli acupoint (ST36) has shown therapeutic potential for sepsis due to its ability to limit inflammation and to regulate gastrointestinal tract symptoms. However, the mechanisms contributing to the effects of EA at ST36 on sepsis and connections with the intestinal flora remain unclear. This study was designed to explore the effects of EA at ST36 on Toll-like receptor 4 signaling and the intestinal flora.

**Methods:**

ICR mice were randomly divided into 4 groups: control group, model group, EA group, and sham EA group. EA at ST36 was performed at 2.5 mA and 2 to 100 Hz, and the 30 min of dense wave was achieved over 5 days. A sepsis model was built by intraperitoneal injection of lipopolysaccharide (LPS, 10 mg/mL). The levels of expression of interleukin-1*β* (IL-1*β*), IL-6, tumor necrosis factor-*α* (TNF-*α*), and IL-10 were detected by enzyme-linked immunosorbent assays, and lactate dehydrogenase (LDH) levels in serum were measured by biochemical tests. Expression levels of Bax, Bcl2, cleaved caspase-3, Toll-like receptor (TLR4), nuclear factor-kappa B (NF-*κ*B), and myeloid differentiation factor 88 (MyD88) were assessed by the Western blotting. Terminal deoxynucleotidyl transferase-mediated dUTP nick-end labeling (TUNEL) staining was used to evaluate apoptosis. The intestinal microecology was assessed via 16S rRNA gene sequencing.

**Results:**

EA at ST36 reduced the expression of IL-1*β*, IL-6, and TNF-*α* and increased the expression of IL-10 to inhibit the inflammatory response. EA at ST36 also inhibited apoptosis, as measured by TUNEL staining, and decreased the Bax/Bcl2 ratio and levels of caspase-3 and cleaved caspase-3, as well as LDH release. Our results suggest that alleviation of sepsis may correlate with the downregulation of levels of TLR4, NF-*κ*B, and MyD88. Importantly, EA at ST36 improved the diversity of the intestinal flora and increased the abundance of Firmicutes and Actinobacteria. Conclusion. EA at ST36 prevented sepsis from worsening by inhibiting inflammation and apoptosis, which correlated with the regulation of the TLR4/NF-*κ*B/MyD88 signaling axis and modulation of the intestinal flora.

## 1. Introduction

Sepsis, a complex syndrome caused by a host-mediated inflammatory response during infection, is associated with high morbidity and mortality and is an expensive medical issue [1, 2]. The core mechanism leading to the complications of sepsis is inflammation [3]. Many factors can trigger sepsis, including lipopolysaccharide (LPS), the main molecular component of the outer membrane of Gram-negative bacteria. The release of LPS is typically associated with the induction of a massive release of pro-inflammatory cytokines, leading to an uncontrolled inflammatory response followed by systemic inflammation reaction syndrome (SIRS) [4]. Thus, sepsis and SIRS are different phases of the same pathological process [5].

Toll-like receptor 4 (TLR4) is a key target in the pathophysiology of sepsis. This receptor can recognize LPS and is responsible for initiating the inflammatory cascade, suggesting the potential value of targeting TLR4 in the treatment of sepsis [6]. In addition, accumulating evidence shows that patients who suffer from sepsis often have disrupted intestinal flora [7]. Notably, disorders of the intestinal flora are known to lead to inflammatory responses, and thus, they are able to promote the septic response [8]. In recent years, great advances in therapy and management have helped infection control and improved the quality of life of many patients, but sepsis remains a critical issue due to high mortality rates and the negative side effects of current therapies [2, 9]. Accordingly, identifying new therapies to control the extreme inflammatory status in sepsis by focusing on TLR4 signaling and intestinal flora is an important research avenue.

Acupuncture is a characteristic therapy in traditional Chinese medicine (TCM), and it has been used widely in China to treat sepsis for many years [10]. One form of this therapy, electroacupuncture (EA), stimulates acupoints with filiform needles inserted at specific points on the body [11]. Zusanli (stomach meridian, ST36) is an acupoint located 3 cm below the knee joint on the anterior aspect of the leg. ST36 is a potential acupoint of importance in the treatment of sepsis due to its ability to control inflammation and to regulate aspects of the gastrointestinal tract [12]. For example, a randomized controlled trial reported that EA at ST36 reduced inflammatory responses and improved intestinal dysfunction in sepsis patients [13]. However, the mechanisms whereby EA at ST36 regulated inflammation and intestinal flora to inhibit the progression of sepsis remain unclear. Therefore, in this study, a model of sepsis induced by LPS was constructed to explore the effects of EA at ST36 on TLR4 signaling and the intestinal flora.

## 2. Materials and Methods

### 2.1. Animals

Male Institute of *Cancer* Research (ICR) mice (body weight: 20 ± 2 g; age: 6–8 weeks) were bought from Zhejiang Experimental Animal Center (SYXK (Zhe) 2016–0022) and housed in the Laboratory Animal Center of Zhejiang Academy of Traditional Chinese Medicine at 22°C with a 23 h of light/dark cycle. All animals were housed and cared for according to the Chinese Pharmacological Society Guidelines for Animal Use. The work was approved by the Committee on the Ethics of Zhejiang Academy of Traditional Chinese Medicine (Permit Number: 2019–059).

### 2.2. Experiment Grouping and Treatments

After 7 d of adaptive feeding, 40 ICR mice were randomly divided into 4 groups: control group (*n* = 10), model group (*n* = 10), EA group (*n* = 10), and sham EA group (*n* = 10). Mice of the EA group were subjected to EA at ST36 for 5 days as follows. An acupuncture needle (0.25 × 0.25 mm, Wujiang Jiachen Acupuncture Instrument Co., Ltd, Jiangsu, China) was inserted perpendicularly at a depth of 3 mm into the bilateral Zusanli acupoints (ST36), located in the posterolateral part of the knee joint and approximately 3.5 mm below the small head of the fibula as described in Xinghuabang acupuncture points commonly used in mice. Both output leads from a programmable electroacupuncture stimulator (Suzhou Medical Supplies Co., Jiangsu, China) were connected to the handles of the needles. EA was applied for 30 min each day, with a dense wave intensity of 2.5 mA and 2–100 Hz.

Mice of the sham EA group had needles inserted perpendicularly at non-acupoints, located 5 mm from the Zusanli acupoints, and the other operations were the same as for the EA group. Control and model group mice did not receive EA treatment. On day 5, mice from all groups except the control group received LPS (10 mg/mL, Sigma, Shanghai, China) by intraperitoneal injection to a build sepsis model after the final intervention treatment. The control group was treated with an equivalent volume of saline. After 3 h, all mice were sacrificed to collect blood, colon tissue, and feces. These samples were stored at -80°C, and the ileum tissue was placed in formalin.

### 2.3. Enzyme-Linked Immunosorbent Assay (ELISA)

ELISA kits were used to measure the serum levels of IL-1*β* (Cat. #m11063132-C, Shanghai Enzyme Linked Immunobiology Co., Ltd, Shanghai, China), IL-6 (Cat. #m1002293-C, Shanghai Enzyme Linked Immunobiology Co., Ltd, Shanghai, China), IL-10 (Cat. #m1002285-C, Shanghai Enzyme Linked Immunobiology Co., Ltd, Shanghai, China), and TNF-*α* (Cat. #m1002095-C, Shanghai Enzyme Linked Immunobiology Co., Ltd, Shanghai, China).

### 2.4. Enzyme Assay

The serum levels of lactate dehydrogenase (LDH) were detected with a kit (A202-2, Nanjing Jiancheng Bioengineering Institute, Nanjing, China) that was used according to the manufacturer's instructions. Briefly, samples (20 *μ*L) were incubated with 5 *μ*L distilled water and 25 *μ*L matrix buffer for 15 min at 37°C and then incubated with 25 *μ*L 2, 4-dinitrophenyl-hydrazone for another 15 min at 37°C. Next, NaOH (0.4 mol/L, 250 *μ*L) was added, and the mixture was placed at room temperature for 5 min. Finally, OD values at the wavelength of 450 nm were measured with a microplate reader (Epoch, BioTek Instruments, Inc., Vermont, USA).

### 2.5. Western Blot

Total protein was extracted from 80 mg samples of colon tissue with 400 *μ*L of radioimmunoprecipitation assay buffer (Solarbio, Beijing, China). Proteins (10 *μ*g) were separated using SDS-polyacrylamide gel electrophoresis (10% gels) and then were transferred to polyvinylidene fluoride membranes. The membranes were blocked in 5% skim milk for 2 hours and incubated with one of the following primary antibodies overnight at 4°C : TLR4 (1 : 1000, 19811-1-AP, Proteintech, Wuhan, China), MyD88 (1 : 2000, AF5195, Affinity Bioscience, Beijing, China), NF-*κ*B (1 : 2000, 10745-1-AP, Proteintech, Wuhan, China), p–NF–*κ*B (1 : 2000, AF3387, Affinity Bioscience, Beijing, China), cleaved caspase-3 (19677-1-1AP, 1 : 2000, Proteintech, Wuhan, China), Bcl2 (1 : 3000, 26593-1-AP, Proteintech, Wuhan, China), Bax (1 : 12,000, 50599-2-Ig, Proteintech, Wuhan, China), or *β*-actin (1 : 50,000, 6609-1-Ig, Proteintech, Wuhan, China). On the next day, the membranes were incubated with horseradish peroxidase-conjugated goat anti-rabbit IgG (*H* + *L*) (1 : 10000, Affinity Bioscience, Beijing, China) or goat anti-mouse IgG (*H* + *L*) (1 : 10000, SA00001-1, Affinity Bioscience, Beijing, China) for 2 hours at room temperature. An ECL Chemiluminescent Kit (Beyotime, Shanghai, China) was utilized to detect the protein bands. Gel-Pro Analyzer software (Version: 4.0.0.4) was used to quantify the band intensities.

### 2.6. Terminal Deoxynucleotidyl Transferase-Mediated dUTP Nick-End Labeling (TUNEL) Staining

Ileum tissue (4 *μ*m) embedded in paraffin was dewaxed with xylene and dehydrated with ethanol. The section was incubated with protease K (20 *μ*g/mL, Beyotime, Shanghai, China) for 30 min at 37°C. After removing the protease K with PBS washes, the section was incubated with 50 *μ*L TUNEL decoction liquid for 60 min at 37°C in the dark. Finally, nuclei were counterstained with DAPI, and the section was observed under a fluorescence microscope (Nikon, Japan).

### 2.7. Sequencing of 16S rRNA

Microbial community genomic DNA was extracted from samples of feces using the E.Z.N.A® Soil DNA Kit (Omega BioTek, Norcross, GA, USA) according to manufacturer's instructions. The concentrations and purity were determined with a NanoDrop 2000 UV-Vis spectrophotometer (Thermo Scientific, Wilmington, USA). The hypervariable regions V3–V4 of the bacterial 16S rRNA gene were amplified with primer pairs 338F (5′-ACTCCTACGGGAGGCAGCAG-3') and 806R (5′-GGACTACHVGGGTWTCTAAT-3′) with an ABI GeneAmp® 9700 PCR thermocycler (ABI, CA, USA). The PCR conditions were as follows: initial denaturation of 95°C for 3 min; 27 cycles of 95°C denaturation for 30 s, 55°C annealing for 30 s, and 72°C extension for 30 s; and a final 72°C extension for 10 min. Sequencing was performed using Illumina's MiSeq PE300 platform (Shanghai Majorbio Technology Co., Ltd).

### 2.8. Statistical Analysis

Data are expressed as the mean ± standard deviation and were analyzed by SPSS 25.0 and GraphPad Prism 8.0.1 software. One-way ANOVA was used for comparisons among groups, and the least significant difference (LSD) t was used for further comparison between the two groups. The microbial diversity parameters and composition analyses were evaluated using the Kruskal–Wallis test. *P* value < 0.05 was considered statistically significant.

## 3. Results

### 3.1. The Effects of EA at ST36 on the Inflammatory Response in Septic Mice

**Figure 1:**
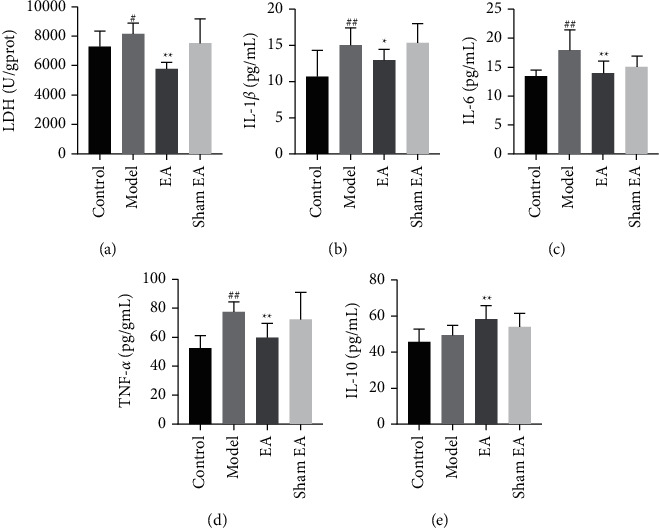
EA at ST36 suppressed the inflammatory response in septic mice. The concentrations of (a) LDH, (b) IL-1*β*, (c) IL-6, (d) TNF-*α*, and (e) IL-10 in serum were detected by ELISA. Data are expressed as mean ± SD. ^##^*P* < 0.01 compared with the control group; ^*∗∗*^*P* < 0.01 and ^*∗*^*P* < 0.01 compared with the model group. The effect of EA at ST36 on apoptosis in cells of septic mice.

The impact of EA at ST36 on the production of LDH and the levels of IL-1*β*, IL-6, TNF-*α*, and IL-10 in each group are presented in Figure 1. The serum LDH level in the model group was significantly higher than in the control group (*P* < 0.01), and it was significantly lower in the EA group than in the control group (*P* < 0.05, Figure 1(a)). In addition, as compared with mice in the control group, mice in the model group exhibited a significant increase in the levels of IL-1*β*, IL-6, and TNF-*α* (*P* < 0.01, Figure 1(b)–1(d)). Compared with mice of the model group, EA group mice had significantly lower IL-1*β*, IL-6, and TNF-*α* levels (*P* < 0.01, 0.05) and a significantly higher IL-10 level (*P* < 0.01, Figure 1(e)). The levels of these inflammatory indicators in sham EA mice were not significantly different from those in the model mice (all *P* < 0.05, Figures 1(a)–1(d). These results suggested that EA at ST36 reduced the inflammatory response in septic mice.

**Figure 2:**
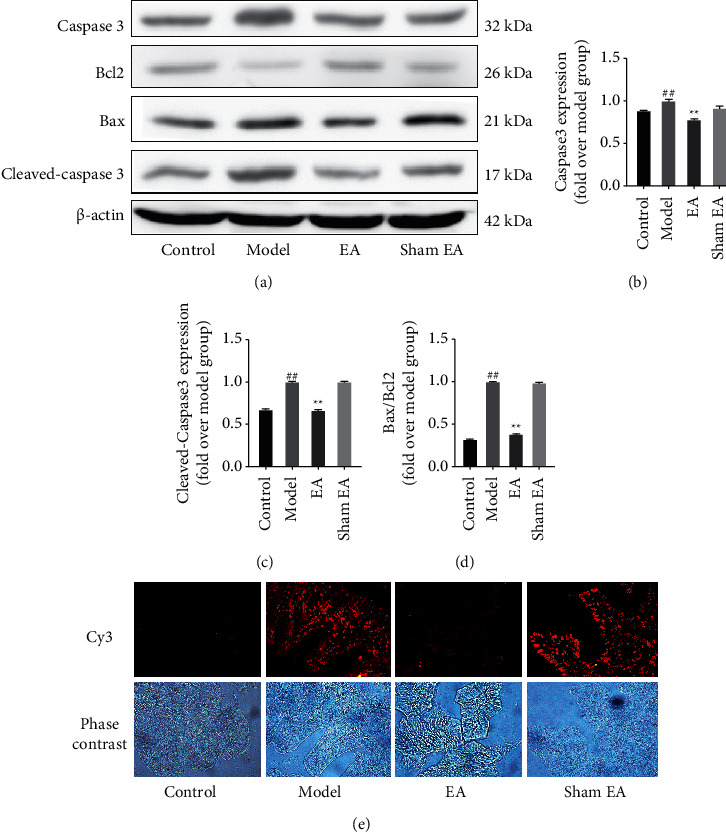
EA at ST36 inhibited apoptosis of cells of the intestinal tissue. (a) The expression levels of caspase 3, cleaved caspase 3, Bcl2, Bax, and *β*-actin were detected by the Western blot. Gel-Pro Analyzer software was used to quantify the levels of expression of (b) caspase 3, (c) cleaved caspase 3, and (d) Bcl2/Bax. (e) A TUNEL assay was used to measure intestinal tissue apoptosis. Cy3 : cyanine 3. Data are expressed as mean ± SD. ^##^*P* < 0.01 compared with the control group; ^*∗∗*^*P* < 0.01 compared with the model group. Modification of signaling through the TLR4/MyD88/NF-*κ*B pathway.

Apoptosis of cells is a key mechanism leading to the symptoms of sepsis. To determine the effect of EA at ST36 on apoptosis in the LPS-induced model of sepsis, the levels of expression of apoptosis-related proteins (Bax, Bcl2, caspase-3, cleaved caspase 3) were quantified (Figure 2(a)). The results showed that model group mice had higher expressions of caspase 3, cleaved caspase 3, and Bax/Bcl2 than did mice of the control group, but pretreatment with EA at ST36 reversed this trend (Figures 2(b)–2(c), *P* < 0.01). Similarly, TUNEL staining indicated higher levels of apoptosis in cells of the intestinal tissue after LPS treatment than in the control group. In sharp contrast, mice pretreated with EA at ST36 prior to exposure to LPS showed little apoptosis in intestinal tissues (Figure 2(d)).

**Figure 3:**
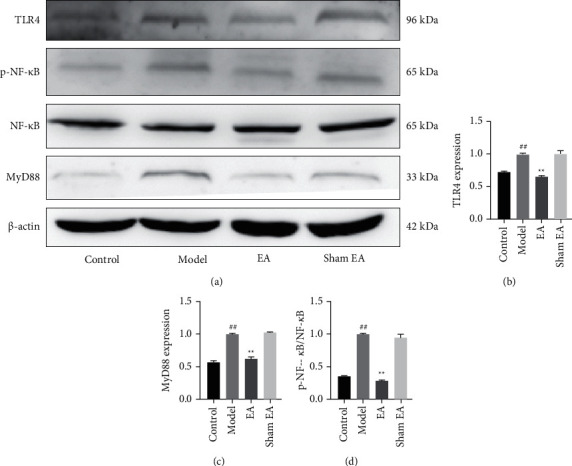
EA at ST36 influenced the TLR4/MyD88/NF-*κ*B pathway. (a) Western blotting was used to detect the levels of expression of TLR4, MyD88, and NF-*κ*B and the phosphorylation of NF-*κ*B (p–NF–*κ*B). Gel-Pro Analyzer software was used to quantify (b) TLR4, (c) MyD88, (d) and NF-*κ*B/p–NF–*κ*B. Data are expressed as mean ± SD. ^##^*P* < 0.01 compared with the control group; ^*∗∗*^*P* < 0.01 compared with the model group.

To investigate the potential mechanism of the effect of EA at ST36 on LPS-induced sepsis, the levels of proteins from intestinal tissue in the TLR4/MyD88/NF-*κ*B pathway were detected by the Western blot (Figure 3(a)). The results showed that the expression levels of TLR4 and MyD88 and the phosphorylation of NF-*κ*B were significantly increased in model group mice, compared with the control group (*P* < 0.01). However, in the EA group, the expressions of TLR4 and MyD88 and phosphorylation of NF-*κ*B were lower than in the model group (*P* < 0.01, Figures 3(b)–3(d). These results suggested that EA at ST36 might downregulate the TLR4/MyD88/NF-*κ*B pathway in this model of LPS-induced sepsis.

### 3.2. Diversity Analysis of the Intestinal Flora

**Figure 4:**
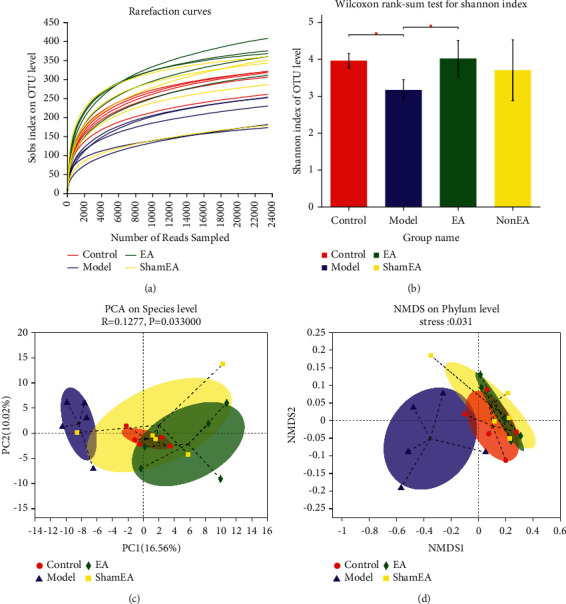
EA at ST36 altered the diversity of the intestinal flora. (a) Rarefaction curves were used to determine whether the amount of sequencing data was sufficient. (b) The differences in diversity of the intestinal flora among groups as noted by alpha diversity. (c) PCA : the closer the two sample points are, the more similar the species composition of the two samples is. (d) NMDS : the closer the sites are, the more similar the species composition of the two samples is. Stress indicates the quality of NMDS analysis results. Generally, it is considered that a stress value less than 0.1 indicates a good sort. OTU: operational taxonomic unit.

After EA treatment and establishment of the sepsis model, the intestinal microbiome of the mice was investigated. DNA was isolated from the feces of the mice, and the 16S rRNA was amplified and sequenced. After subsampling each sample to an equal sequence depth (23,516 reads per sample) and clustering, operational taxonomic units (OTUs) were defined as clusters sharing 97% sequence similarity. The number of OTUs in mice of the four groups ranged from 173 to 408. Good's coverage for the observed OTUs was greater than 99.6%, and the rarefaction curves showed clear asymptotes (Figure 4(a)), which indicated a near-complete sampling of the community. Alpha-diversity analyses demonstrated that LPS induced an increase in the diversity of the intestinal flora in the model group as compared with the control group (Shannon index: 3.16 ± 0.28 vs 3.96 ± 0.20, *P*=0.012). However, the diversity of the intestinal flora in the EA group was higher than that observed in the LPS group (Shannon index: 4.01 ± 0.49 vs 3.16 ± 0.28, *P*=0.036, Figure 4(b)). Principal component analysis (PCA) and nonmetric multidimensional scaling (NMDS) both indicated that the intestinal flora of the four groups were different at the species level, but the composition of the intestinal flora was more similar between the control group and the EA group (PCA : *P*=0.033; NMDS: stress = 0.031, Figures 4(c) and 4(d)).

### 3.3. Variation in the Composition of the Intestinal Flora

**Figure 5:**
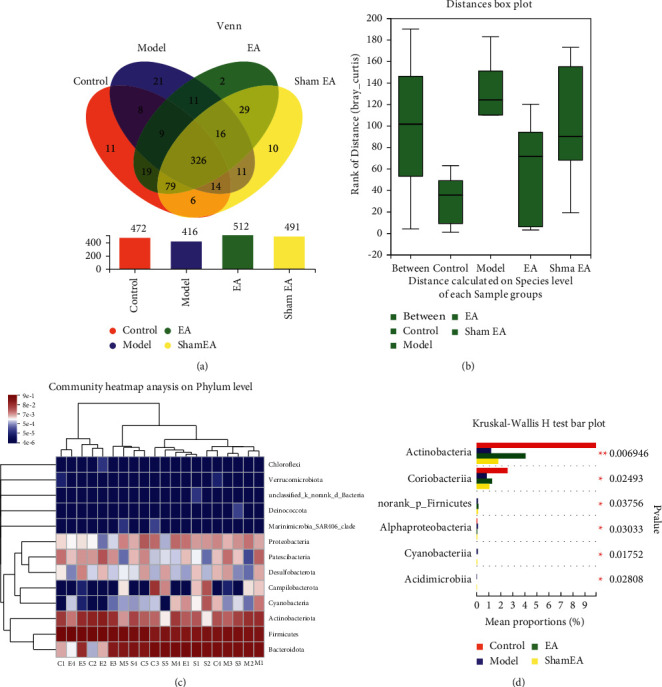
EA at ST36 changed the composition of the intestinal flora. (a) Venn diagram showing the OTUs in each group. (b) Analysis of similarities was used to test whether differences between groups (two or more groups) were significantly greater than differences within groups. (c) A heat map visually indicates the composition of the communities in each sample. C: control; M: model; E: electroacupuncture at Zusanli; S: sham EA; (d) species difference analysis among the four groups.

The control, model, EA, and sham EA groups were found to have 472, 416, 512, and 491 OTUs, respectively. A Venn diagram was established to compare the distribution of these OTUs, and a total of 326 OTUs were identified to be common to the four groups (Figure 5(b)). Analysis of similarities (ANOSIM) revealed significant differences in compositions of intestinal flora among the four groups (*P*=0.005, Figure 5(b)). A heat map was used to display the composition of the intestinal flora on the level of the phylum, and Bacteroidota, Firmicutes, and Actinobacteria were found to be the dominant communities. The control group and the EA group had similar compositions of dominant flora (Figure 5(c)).

To further investigate the dominant flora in each group, a difference analysis was carried out on the intestinal flora on the class level. The results showed that Actinobacteria, Coriobacteriia, Norank_p_Fimicutes, Alphaproteobacteria, Cyanobacteria, and Acidimicrobiia were different among the four groups (*P* < 0.05, 0.01). The relative abundance of Actinobacteria and Coriobacteriia in the model group was lower than in the control and EA groups. The relative abundance of Norank_p_Fimicutes was higher in the EA group than in the model group (Figure 5(d)).

### 3.4. Variation of Biomarkers in the Intestinal Flora

**Figure 6:**
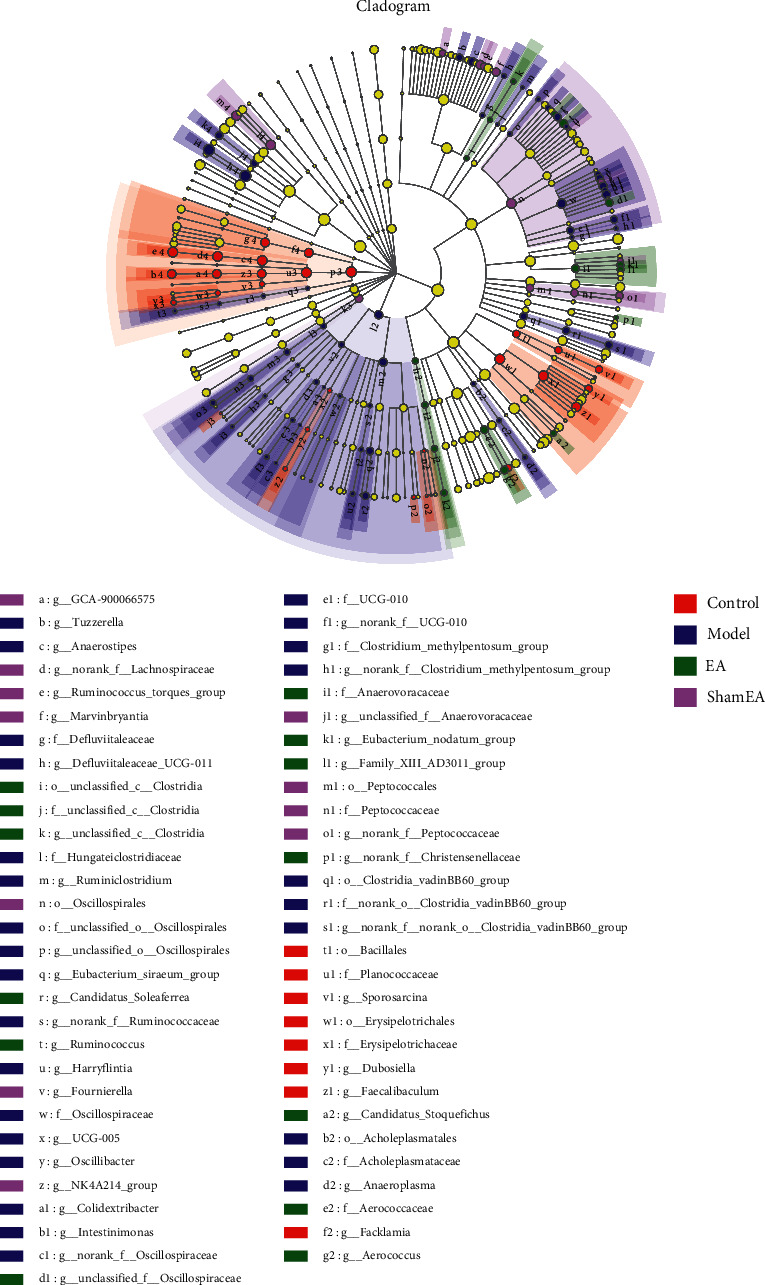
Cladogram displaying the LDA effect size analysis. Different color nodes represent the microbial groups that were significantly enriched in the corresponding groups of mice and that were found to have a significant influence on the differences between the groups. The pale yellow nodes indicate the microbial groups that had no significant difference among different groups or had no significant effect on the differences between groups.

LDA effect size (LEfSe) analysis was used to identify biomarkers with significant differences in abundance between groups (LDA value > 2). At the phylum level, Actinobacteriota, Cyanobacteria, and Proteobacteria were found to be enriched in the control group, sham EA group, and EA group, respectively. At the class level, Norank_p_Firmicutes was enriched in the EA group. At the order level, Acholeplasmatales was enriched in the model group, Erysipelotrichales and Bacillales were enriched in the control group, Unclassified_c_Clostridia was enriched in the EA group, and Oscillospirales was enriched in the sham EA group (Figure 6).

## 4. Discussion

Sepsis, a form of SIRS that can result in end-organ damage and potentially death, is an important public health issue [1]. Inflammatory responses play initial and essential roles in the development of sepsis [14]. TNF-*α* released by activated macrophages and neutrophils then further stimulates the release of other inflammatory cytokines, including IL-1*β* and IL-6 [15]. In turn, IL-10 is an anti-inflammatory cytokine that can inhibit the production of TNF-*α*, IL-1*β*, and IL-6 [16]. TCM has been shown to be effective in the treatment of sepsis-associated inflammation. For example, a systematic review and meta-analysis demonstrated that TCM treatment was associated with lower levels of IL-6 and TNF-*α* among sepsis patients [17]. Previous research has also indicated that the stimulation of the Zusanli (ST36) acupoint has anti-inflammatory effects on an inflammatory disease model [18, 19]. The results in this also showed that EA at ST36 inhibited the production of TNF-*α*, IL-1*β*, and IL-6 and increased IL-10 production in septic mice, suggesting its therapeutic effect on sepsis.

LDH is recognized as the maker of inflammation and tissue injury, and its level is positively associated with the severity of sepsis [20]. Here, the data showed that serum LDH in mice treated with LPS was increased compared with mice of the control group, suggesting that the treated mice experience sepsis-induced tissue damage. Therefore, this research indicated that sepsis is not only accompanied by inflammation but also extensive apoptosis, which could induce LDH release due to structural damage to the affected cells [21]. Similarly, the relative levels of Bax, a proapoptotic protein that can release apoptosis-promoting substances into the cytoplasm [22], and Bcl2, an antiapoptotic protein that blocks oligomerization of proapoptotic proteins [23], suggested that sepsis is correlated with increased apoptosis. In addition, we also investigated the expression and activation of caspase-3, and the vital terminal splicing enzyme in the process of apoptosis is one of the executors of apoptosis [24]. As expected, our results suggested that EA at ST36 decreased LDH levels and inhibited apoptosis by lowering the Bax/Bcl2 ratio and caspase as well as cleaved caspase-3 in septic mice. These results correlated with a decrease in cells positive for TUNEL staining.

Since EA at ST36 limits the occurrence and development of sepsis due to anti-inflammation and anti-apoptosis effects, we further investigated its mechanism. TLR4-mediated MyD88/NF-*κ*B signaling way is the key pathway in sepsis pathophysiology [25]. Briefly, LPS interacts with TLR4 to induce the activation of MyD88-dependent intracellular signaling [26]. MyD88 is the essential downstream signaling effector of the TLR4 receptor complex, and it is also an important adapter protein in the NF-*κ*B signaling pathway [27]. Once being activated, the NF-*κ*B p65 subunit translocates into the nucleus, triggering the production of inflammatory factors, including TNF-*α*, IL-6, and IL-1*β* [28]. In our study, phosphorylation of NF-*κ*B and levels of TLR4 and MyD88 proteins in the model group increased after LPS stimulation, but these factors were significantly inhibited by EA at ST36 in the alleviation of sepsis. In agreement with our research, Xv et al. also found that schizandrin B protects against LPS-induced sepsis via interaction with TLR4/MyD88/NF-*κ*B signaling [29]. Therefore, our results imply that TLR4/MyD88/NF-*κ*B signaling may take part in the effect of EA at ST36 against sepsis.

It is known that sepsis patients have dysbiosis of the gut microbiota due to systemic inflammation. In turn, the change in intestinal flora exacerbates the symptoms of sepsis [30]. Accordingly, decreased intestinal flora diversity has been suggested to be one of the major features of sepsis [32]. Previous research reported that mice with increased gut microbiome alpha diversity have an immune system that is primed to respond to sepsis [31]. Similarly, we found that LPS reduced the diversity of the intestinal flora in the model group but that this diversity was improved by pretreatment with EA at ST36.

In addition, variation in certain bacteria is also a hallmark of sepsis. For example, the abundance of Firmicutes and Actinobacteria in sepsis patients has been shown to help restore gut homeostasis [33, 34]. It was reported that the most members of Firmicutes, such as Lactobacillus, are beneficial bacteria that might produce antimicrobial substances to prevent disease development [35], and it is well known that Actinobacteria are the main antibiotic-producing bacteria [36]. We assumed that it might be the reason to fight the sepsis. These beneficial bacteria were depleted upon treatment with LPS, but their abundance was restored with pretreatment with EA at ST36. Taken together, these results suggest that the LPS-induced inflammatory response in a mouse model of sepsis is sufficient to affect microbiome composition. Importantly, EA at ST36 modulates the inflammatory response and changes microbiome composition to resist the development of the disease.

## 5. Conclusion

In summary, our results demonstrated that EA at ST36 alleviated LPS-induced sepsis by inhibiting inflammatory response and apoptosis. In addition, the regulation of sepsis may be correlated with TLR4/MyD88/NF-*κ*B signaling and alterations to the intestinal flora. Our work reveals that EA at ST36 may be a promising option for the treatment of sepsis induced by LPS. However, the further mechanism is needed to be illustrated.

## Data Availability

No data were used to support this study.

## Abbreviation

LPS: Lipopolysaccharide
SIRS: Systemic inflammation reaction syndrome
TLR4: Toll-like receptor 4
EA: Electroacupuncture
TCM: Traditional Chinese medicine
ELISA: Enzyme-linked immunosorbent assay
LDH: Lactate dehydrogenase
NF-*κ*B: Nuclear factor-kappa B
MyD88: Myeloid differentiation factor 88
IL-1*β*: Interleukin-1*β*
IL-6: Interleukin-6
TNF-*α*: Tumor necrosis factor-*α*
IL-10: Interleukin-10.
